# What is the optimal duration of home-video-EEG monitoring for patients with <1 seizure per day? A simulation study

**DOI:** 10.3389/fneur.2022.938294

**Published:** 2022-08-22

**Authors:** Tatiana Vander, Tatiana Stroganova, Diya Doufish, Dawn Eliashiv, Tal Gilboa, Mordekhay Medvedovsky, Dana Ekstein

**Affiliations:** ^1^Herzfeld Geriatric Rehabilitation Medical Center, Gedera, Israel; ^2^The Faculty of Medicine, Hebrew University of Jerusalem, Jerusalem, Israel; ^3^MEG-Center, Moscow State University of Psychology and Education, Moscow, Russia; ^4^Department of Neurology and Agnes Ginges Center for Human Neurogenetics, Hadassah Medical Organization, Jerusalem, Israel; ^5^Department of Neurology, David Geffen School of Medicine, University of California, Los Angeles, Los Angeles, CA, United States; ^6^The Neuropediatric Unit, Division of Pediatrics, Hadassah Medical Organization, Jerusalem, Israel

**Keywords:** drug-resistant epilepsy, seizure frequency, cycling seizures, epilepsy surgery, presurgical evaluation

## Abstract

Ambulatory “at home” video-EEG monitoring (HVEM) may offer a more cost-effective and accessible option as compared to traditional inpatient admissions to epilepsy monitoring units. However, home monitoring may not allow for safe tapering of anti-seizure medications (ASM). As a result, longer periods of monitoring may be necessary to capture a sufficient number of the patients' stereotypic seizures. We aimed to quantitatively estimate the necessary length of HVEM corresponding to various diagnostic scenarios in clinical practice. Using available seizure frequency statistics, we estimated the HVEM duration required to capture one, three, or five seizures on different days, by simulating 100,000 annual time-courses of seizure occurrence in adults and children with more than one and <30 seizures per month (89% of adults and 85% of children). We found that the durations of HVEM needed to record 1, 3, or 5 seizures in 80% of children were 2, 5, and 8 weeks (median 2, 12, and 21 days), respectively, and significantly longer in adults −2, 6, and 10 weeks (median 3, 14, and 26 days; *p* < 10^−10^ for all comparisons). Thus, longer HVEM than currently used is needed for expanding its clinical value from diagnosis of nonepileptic or very frequent epileptic events to a presurgical tool for patients with drug-resistant epilepsy. Technical developments and further studies are warranted.

## Introduction

Video-EEG monitoring (VEM) provides documentation and characterization of epileptic seizures, aids in the diagnosis of non-epileptic events, and is always performed as part of the evaluation of patients with drug-resistant epilepsy (DRE) (1). The common practice is to admit the patient to an epilepsy monitoring unit (EMU) and record several habitual seizures during a relatively short time, of 5–7 days. As to facilitate seizures being captured during this time frame, anti-seizure medications (ASM) are often tapered down(2). However, inpatient admissions to the EMU may be limited due to lack of availability of equipment and beds. Inpatient EMUs are also resource intensive, costly (3), and may carry significant safety issues (4). Thus, VEM, being the cornerstone of DRE assessment, slows the evaluation of patients with DRE in developed countries and may not be always available for DRE patients in many developing countries (5).

The alternative to inpatient VEM is an outpatient home VEM (HVEM). Today, most HVEM recordings are limited to several days (6, 7), due to the need for adhesion of the electrodes to the scalp by trained technicians in EEG laboratories, at least every 3 days. Because ASM reduction at home is unsafe, the likelihood of capturing seizures depends on the seizure frequency in each patient. It was recently reported that more than 97% of first clinical events and more than 95% of the mean number of subsequent clinical events were observed in adult and pediatric patients during 72 and 48 h of HVEM, respectively (8). However, most captured events in this study were non-epileptic (24.8 times more than epileptic in adults and 10 times more in children). The optimal duration of HVEM needed for recording true epileptic seizures remains unknown.

The current study aimed to evaluate how much time is needed to record epileptic seizures by HVEM. We posed goals of capturing one, three, or five seizures on different days, corresponding to different clinical needs of video-EEG varying from just discrimination of epileptic/non-epileptic events to recording a variety of non-clustered seizures required for reasonable exclusion of multiple epileptic foci (9, 10). We based our evaluation on natural seizure frequencies reported in a large cohort of more than a million seizures logged by 10,186 adult and pediatric patients in a large electronic seizure diary (11). However, we excluded patients with an average seizure frequency of one seizure per day or more, obviously not posing a significant challenge for seizure capture with either VEM or HVEM, and patients with very rare seizures (<1 per month). In adults, we also considered temporal cycling of seizures, as reported for patients with focal DRE who were continuously monitored with intracranial EEG for long periods of time (12). To simulate seizures' capture by HVEM, we constructed synthetic annual time-courses of seizure occurrence in a big synthetic cohort of 100,000 pediatric and adult patients with less than one seizure per day.

## Method

**Figure 1:**
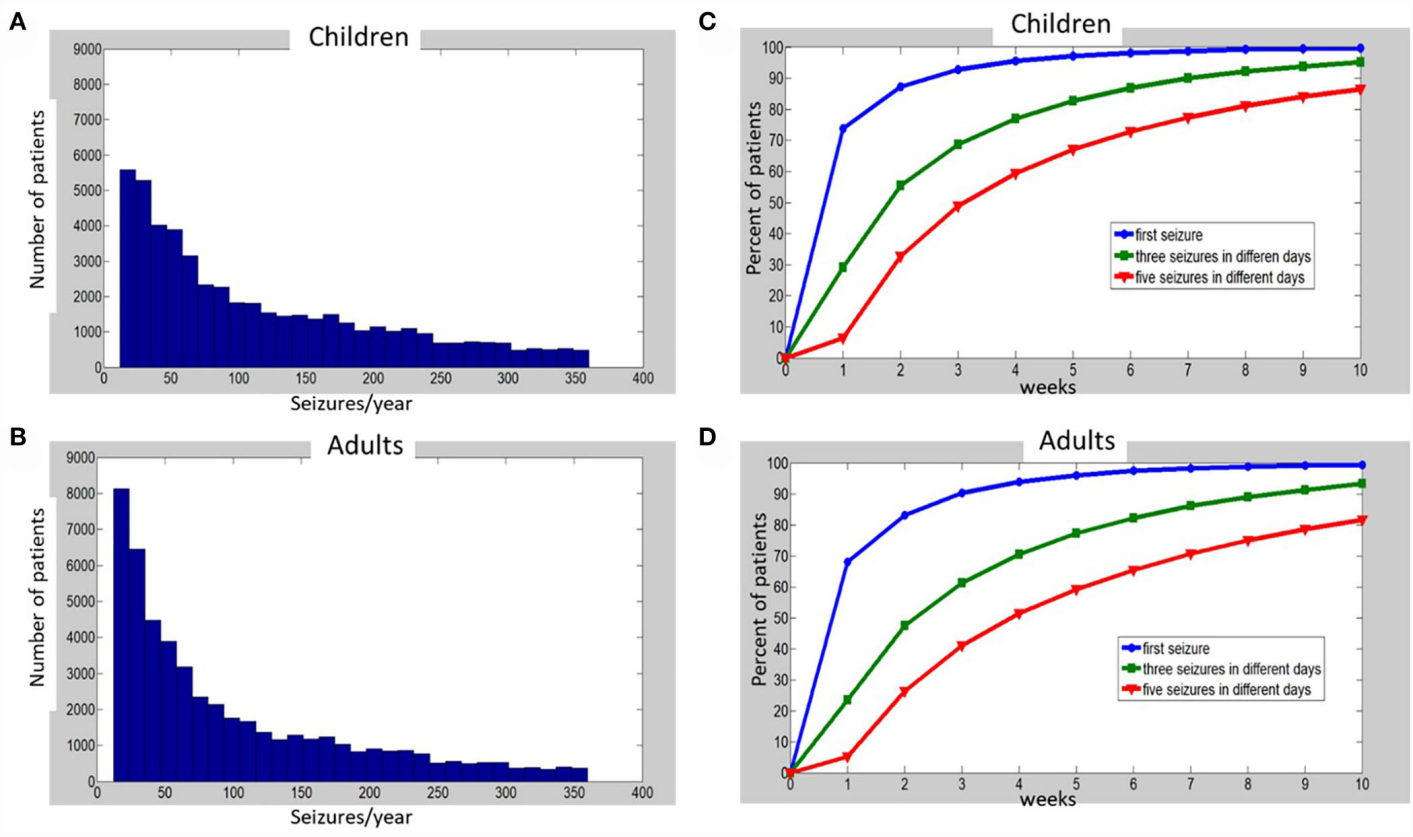
Histograms of simulated annual seizure frequencies distribution among 50,000 children **(A)** and 50,000 adults **(B)** with epilepsy, and percentages of patients (C—children, D—adults), for which one (blue), three (green), or five (red) seizures on different days were captured at the end of each week, up to 10 weeks of monitoring. See Supplementary material for details of simulations and Supplementary Tables S1, S2 for the raw data that were used to build the charts **(C,D)**.

We simulated daily seizure distribution for 1 year in 50,000 adults and 50,000 pediatric epilepsy patients (Figures 1A,B), and then simulated HVEM sessions for all the patients. We used the Monte Carlo method to combine data from two separate datasets (11, 12). Standard computation time limited the number of simulated time-courses. The MATLAB R2020b (MathWorks) software was used for all simulations.

The information on seizure frequency distribution was taken from Ferastraoaru et al. (11), where data from the Seizure Tracker dataset (www.seizuretacker.com) were analyzed and presented. This dataset is obtained by voluntary and anonymous smartphone-application-based patients' (or their family members') reports of the time of the seizures, rescue medications, VNS magnet swipes, event triggers, and event/post-event descriptions. Patients are also offered to upload video captures of their seizures. We disregarded seizure frequencies below one per month and above 30 per month (observed in 15% of children and 11% of adults in the dataset). Cycling of seizures was reported by Ferestraoaru et al. and also by others who analyzed part of the Seizure Tracker dataset (13). However, given the limitations of self-reported seizures' diaries (14), we decided to use cycling data obtained from long-term intracranial monitoring, a more objective available resource (12). Unfortunately, the information on objective seizure frequency is scarce and relates only to a very specific subset of patients implanted with invasive EEG devices. Thus, we had to combine the data from the two datasets (11, 12) to estimate a seizure distribution as close as possible to the actual real patients' situation. We considered all children and 40% of adults to have randomly distributed seizures over all days of the year, whereas 60% of time-courses of adult patients were simulated to represent multidien cycling of seizures. Among the cycling seizures time-courses, 20% were simulated as 7-days cycles, 17% as 15-days, 30% as 20-days, and 33% as 30 days cycles (12), as further elaborated in Section Methods, representing the second step of simulations.

We performed the simulations in three sequential steps:

1. Distribution of annual seizure frequency among simulated patients.
2. Generation of daily seizure distribution for 1 year in individual simulated patients.
3. Home video EEG monitoring (HVEM) simulation.

### Distribution of annual seizure frequency among simulated patients

The goal of this stage was to generate a realistic distribution of annual seizure frequency among a group of simulated epilepsy patients (50,000 adults and 50,000 children), who have at least one seizure per month and no more than 30 seizures per month. We used the probability density function depicted in Supplementary Figure S1 of Ferastoauru et al. (11), and made the calculations in several steps as follows:

We printed Supplementary Figure S1 from Ferastoauru et al. (11) and manually projected several points on the *X-*axis to the *Y-*axis. *X-*axis (monthly seizure frequency) is the argument of a function and *Y-*axis (probability density) is a function. The following points on *X-*axis were chosen for projection to *Y-*axis: 1, 2, 3, 4, 5, 6, 7, 8, 9, 10, 15, 20, 25, 30 (seizures/month), so that between 1 and 10 seizures/month, steps of 1 were taken and between 10 and 30 seizures/month—steps of 5. The point probability densities corresponding to the chosen *X-*axis points are presented in Supplementary Table S1 (Supplementary material).

These obtained values are the point probability values and therefore represent only probability of the given point on the *X-*axis. To calculate how many patients out of the whole group have a given seizure frequency, it is important to define seizure frequency intervals (on the *X-*axis). We introduced intervals between predefined *X-*axis points to calculate the mean probability density *mPD*_*i*_ for every seizure frequency interval *i*. However, because the function is not linear (in short intervals it can be considered as close to linear), we calculated not exact mean values, but rather some surrogate mean probability density values mPD^*^_i_ by summation of probability densities of the beginning and the end of the interval divided by two.


$$mPD^{*}{\;}_{i}=(PD_{is}+PD_{ie})/2$$

Where mPD^*^_i_ is surrogate mean probability density for seizure frequency interval *i*; *PD*_*is*_ and *PD*_*ie*_ are probability densities for start and end of seizure frequency interval *i*.

The probability that a given patient will have their seizure frequency in a specific interval depends on the mean probability density for this interval and the length of the interval. Therefore, the probability that a given patient has the seizure frequency in a given interval *i* can be expressed as the area under the curve (AUC) of interval *i* of the probability density function, which was calculated as:


$$P_{i}=mPD^{*}{\;}_{i}\;L_{i}$$

Where *P*_*i*_ is the probability that a patient has seizure frequency in interval *i*; *L*_*i*_ is the length of interval *i* (number of seizure frequency units [seizure/month] in interval *i*).

The probability densities in Supplementary Figure S1 of Ferastoauru et al. correspond to the whole population of patients with epilepsy, while in our study we limited the population to patients who have from 1 seizure/month to 30 seizures/month. Therefore, the probabilities that we calculated should be normalized according to the population in our study. Considering that the sum of probabilities for the whole population is 1, we normalized probabilities in every individual interval *i* (probability *i*) as:


$$nP_{i}=P_{i}/\sum_{i=1}^{n}P_{i}$$

Where *nP*_*i*_ is normalized probability that a patient has seizure frequency in interval *i*; *n*—number of seizure frequency intervals.

Next, we generated a random distribution of all simulated patients: 50,000 adults and 50,000 children (using *randi* function of MATLAB to generate an equal random distribution of integers) between the start and the end of each *X-*axis interval (after its edges values were multiplied by 12, transforming the monthly distribution of seizures to an annual one). For example, to generate individual annual seizure frequencies for the patients with between 2 and 3 seizures/month, the *randi* function was applied between 24 and 36 seizures/year. The histograms of the obtained annual distributions are depicted in Figure 1 for children (A) and adults (B). When the characteristics of the distributions (see Figure 1 legend) were translated to monthly seizure frequency (for comparison to Ferastoauru et al. (11)), we obtained a monthly distribution for children with a mean of 9.64 seizures/month (median 7.08, SD 7.61) and one for adults with a mean of 8.3 seizures/month (median 5.5, SD 7.22).

Finally, to obtain a random mixing of patients with various frequencies of seizures, we randomized all patients between different probability density intervals using *randperm* function of MATLAB, performing random permutations between values.

### Generation of daily seizure distribution for 1 year in individual simulated patients

In the previous stage, we defined the distribution of annual seizure frequencies in the adult and the pediatric population of patients with epilepsy. The goal of the present stage was to simulate the daily distribution of seizures in patients with epilepsy. Here we considered two ways of daily seizure distribution:

a. Random distribution with equal probability of seizure occurrence in days throughout the year.
b. Cycling of the seizures: higher probability of seizure in days belonging to the active part of the cycle.

Seizure cycling was reported and quantified in adults from long-term invasive recording devices (12). Our simulation considered the reported seizure multidien cycling, that is, in a scale of week to month, with a mean and standard deviation of phase-locking value (PLV), of 0.34 and 0.18, respectively (12). Because this type of cycling was found in 60% of patients, we divided all adult time-courses into two groups: without cycling (40% of all time-courses) and with cycling (60%). All time-courses of children were simulated without cycling.

a. The time-courses without cycling were constructed in the following way:

After obtaining the total number of annual seizures for each patient, we simulated their distribution throughout 365 days of the year by randomizing seizures using *rand* function of MATLAB, which generates equally distributed pseudorandom numbers between 0 and 1. Occurrence of more than one seizure per day was allowed.

b. The time-courses with cycling were constructed in the following way:

First, we defined the number of seizures in 1 year for individual patients, as described above. Next, we constructed binary time-courses (half cycle—zeros and another half—ones) according to cycles of 7 (20% of all patients with cycling seizures), 15 (17% of the patients), 20 (30%), and 30 days (33%), all starting on day 1 of the time-course with the ones-half-cycle. Since the resolution of time-courses was 1 day, the cycles of 7 and 15 days were divided unevenly: the 7-day cycle had 4 ones and 3 zeros and the 15-day cycle-−7 ones and 8 zeros. Then, we randomly distributed 34 ± 18% (as per the PLV) of seizures between days marked by ones but not by zeros. The rest of the seizures (66 ± 18%) were distributed throughout the 365 days of the same individual time-courses with equal probability for all days.

Thus, in the adult time-courses with cycles (60% of all time-courses), the probability of seizure in the active phase of cycles was 1.34 ± 0.18 times higher than in the inactive phase, while in the time-courses without cycling, the probability of seizure was equal for all days throughout the time-course.

### HVEM simulation

We used two ways of HVEM start randomization. According to the first way, applied only to adults and compared between patients with a cycling course of the disease and without cycling, to simulate the situation with HVEM onset being synchronized with seizure cycles, we added *round(randn(1))* to day 180 of every time-course. Thus, we created a Gaussian distribution of HVEM starting around day 180 with a standard deviation of 1 day. We choose day 180, as it is both the beginning of cycles of 15, 20, and 30 days, and is situated approximately in the middle of the annual time-course. The cycle of 7 days was not accounted for in this distribution, but due to its short duration, it is anyhow less relevant for a precise attempt to capture it.

In the second way, applied to adults and children, the day of HVEM onset was randomized using the rand function of MATLAB by addition of round (rand^*^60) to day 150. Thus, the HVEM onset was unrelated to the onset of seizure cycles, beginning with equal probability in any of the days 151–210 of the time-course. Days 150 + 60 were chosen to ensure the overall similar probability of remaining seizures as for the first way of randomization, which began around day 180, for comparison matters.

The HVEM stopped after achieving the goal of 1, 3, or 5 seizures recording on different days. In this study, we obtained six vectors with 50,000 entries of everyone, corresponding to three clinical goals (1, 2, and 3 seizures on different days) for two age groups (children and adults). Every entry in these vectors is the number of days required to achieve a specific clinical goal for one patient.

As a result of the simulations, we obtained the number of days of HVEM needed to capture 1, 3, and 5 seizures on different days for each of the 50,000 children and 50,000 adult patients. Then, we calculated the descriptive statistics for the recording duration (in days) that was required to fulfill the respective HVEM goals for patients in each age group. In addition, we calculated the percent of patients who achieved a certain clinical goal after every week of continuous HVEM, from week 1 to 10.

### Statistical analyses

Recording durations to the first, three, and five seizures captured on different days were characterized per cohort by calculating the mean (M), standard deviation (SD), median (Md), interquartile range (IQR), and skewness of distribution. We compared the number of days required to achieve the different clinical goals between adults and children, and between adults with and without seizure cycling. We used parametric statistics for comparison between the groups, given the very large sample sizes. Two-tailed Student's *t*-test (two-sample *t*-test was used when the sizes of compared populations were different) was performed to assess significant differences between groups and *p* < 0.05 was considered significant.

## Results

The percentages of patients with one, three, or five recorded seizures over time are depicted in Figure 1C, Supplementary Table S2 for children and Figure 1D, Supplementary Table S3 for adults. One week of HVEM resulted in the acquisition of the first seizure in approximately 70% of patients in our simulated cohorts. However, during the first week of HVEM monitoring only a quarter of the patients achieved the goal of three seizures on different days, while the five seizures goal was completed in less than 10% of children and adults (Figures 1C,D; Supplementary Tables S2, S3). The duration of HVEM required to record one, three, or five seizures in 80% of children were 2, 5, and 8 weeks, respectively (Figure 1C; Supplementary Table S2), and 2, 6, and 10 weeks in adults (Figure 1D;  Supplementary Table S3).

Overall, there was a significant difference between adults and children in the time needed to achieve the HVEM goals. Adults required a median of 1 day more than children to record a single seizure, 2 days more for three, and 5 days more than children for capturing five seizures on different days (Table 1).

**Table 1:** Comparison of necessary duration of monitoring between various patient populations (expressed in days).

	**Recording of the first seizure**		***p*-value**	**Recording of 3 seizures on different days**		***p*-value**	**Recording of 5 seizures on different days**		***p*-value**
	**Comparisons between children and adults**								
**Population**	**Adults**	**Children**		**Adults**	**Children**		**Adults**	**Children**	
Mean (median) SD [skewness, IQR]	7.46 (3) 11.98 [3.54, 8]	6.11 (2) 10.54 [3.98, 6]	<10^−10^	23.97 (14) 25.35 [2.14, 25]	20.21 (12) 22.92 [2.53, 20]	<10^−10^	40.62 (26) 37.95 [1.61, 42]	34.25 (21) 34.03 [1.94, 34]	<10^−10^
	**Comparisons between adults with and without seizure cycling**								
**Seizure cycling**	**With**	**Without**		**With**	**Without**		**With**	**Without**	
Adults- random start of HVEM Mean (median) SD	7.58 (3) 12.04	7.27 (3) 11.73	0.0298	24.18 (14) 25.52	23.64 (15) 25.10	0.0196	40.72 (27) 38.22	40.03 (26) 37.27	0.1360
Adults- HVEM start at cycle beginning Mean (median) SD	6.38 (3) 10.86	7.28 (3) 11.86	1.82* 10^−10^	22.69 (12) 26.27	23.99 (14) 25.77	4.2* 10^−5^	39.03 (25) 38.35	40.38 (26) 37.98	0.0036

There was minimal difference in needed HVEM duration between patients with cyclical and non-cyclical seizure patterns, if HVEM was started randomly with respect to the beginning of the cycles (Table 1). However, starting the HVEM within 1 day from the beginning of a cycle represented a significant advantage for the cycling-seizures group, especially for capturing three seizures on different days. The median difference for achieving this goal was 2 days less in adults with cyclical seizure patterns than in those without cycling (Table 1).

## Discussion

The results of our simulation study show that 1 week of HVEM is insufficient to capture enough seizures for presurgical evaluation in the vast majority of patients with an average seizure frequency of <1 per day. This is in line with data from patients implanted with bilateral mesial temporal responsive neurostimulation devices, showing that the average time to capture a seizure originating from the temporal lobe contralateral to the originally active one was 41.6 days (15). Similarly, Syed et al. (16) demonstrated that HVEM of a median of 3 days captured representative events in only about a third of the children and adults included, representing half of the events recorded with inpatient VEM. Furthermore, only 10.6% of patients with interictal activity had also epileptic seizures (in addition to 0.5% with patients with ictal signal only), illustrating the difficulty of capturing genuine epileptic seizures during short-term HVEM (16).

In adults with a cyclical seizure pattern, timing the HVEM study within 1 day of the beginning of a cycle may decrease the total duration of monitoring necessary to capture three non-clustered seizures on 3 different days. Patients with predictable seizure patterns may benefit from intermittent, on-demand HVEM, favoring the “active” days, as revealed by careful seizure history taking and by evaluation of routinely used seizure diaries.

Our simulation study highlights the need for patients' stratification in the clinical use of HVEM and judicious interpretation of HVEM results. Thus, recordings lasting up to 1 week may be suitable merely for diagnosing patients with nonepileptic events and for evaluating patients with daily seizures (8). However, according to our results, it may take an HVEM study five times longer to capture sufficient seizures as part of a presurgical epilepsy evaluation. Likewise, longer than 1-week recordings may be required for evaluating patients with a mixed, epileptic and nonepileptic, seizure disorder. Since often a nonepileptic event is captured before the recording of an epileptic seizure (17), the results of a short HVEM study may ultimately prove as misleading.

Some of the technical challenges posed by the need for longer HVEM may be solved by using electrodes that do not need frequent technician support, for example, dry electrodes (18) or implanted subcutaneous electrodes (19, 20). However, an ideal ultra–long-term HVEM system should provide non-invasive, easily self-applicable electrode arrays, automatic identification of electrode locations and signal quality, offer continuous remote support, and use reliable seizure and spike detection software. While several weeks of full-head-coverage HVEM are technologically possible already nowadays, the clinical implementation of these methods is still limited, or restricted to systems applying only a small number of electrodes (7). Thus, more efforts should be made to transfer technological advancements into practical applications. Such efforts are worthwhile, not only to increase the availability of VEM and to reduce its costs (3) but also to provide a more unbiased picture of the seizure activity, without ASM changes, in the natural environment of the individual patient.

Another important issue to consider on the path of switching in-hospital EMU admissions to HVEM is the daily life practicality of performing prolonged monitoring. While some normal life routine interference is unavoidable using very long HVEM, several strategies can be used to minimize it. For example, recording home VEEG with long interruptions: for example, 1 week recording followed by 1 week interruption and so on, or recording mainly during weekends. For patients with mostly night seizures, overnight recordings with daytime interruptions can be appropriate, while patients with predictable cycling or clustering of seizures may schedule the recordings for the most susceptible times. Thus, the recording strategy should be individually tailored for each patient to fit both their epilepsy characteristics and lifestyle.

The main inherent limitation of this report resides in its design as a simulation study, as well as in relying on a self-reported seizures dataset. As it is known that self-reported diaries result in a lower number of seizures than actually occurring (14), this could have overestimated the length of HVEM needed for capturing seizures. However, self-reported diaries could also include non-epileptic events, as well as auras and other EEG-undetectable seizures. Moreover, part of the recorded data are many times not usable for analysis due to technical issues related to the quality of the EEG and video recordings and patients may opt to use the HVEM in an alternating pattern to mitigate disruption to normal life routine. Thus, overall, in clinical practice, our estimation of the needed length of HVEM may prove correct or even too short, especially for patients with lower seizure frequencies. Another limitation of our study is that cycling was only available for adults, although it may also occur in children. For example, catamenial epilepsy cycling can appear at menarche (21). Prospective studies of reliable EEG recording-enriched seizure prevalence and patterns of their appearance could at least in part address these limitations. However, since the best way to obtain such registries would be through HVEM technologies, a vicious cycle problem is obvious. Therefore, meanwhile, relying on large real-life-based datasets of seizures seems an acceptable compromise. In this study, we did not account for the capture of interictal epileptic activity, which may be of clinical importance, albeit sometimes of equivocal significance. Since interictal activity may prove to be of important diagnostic value, its presence may enable the shortening of HVEM sessions in selected patients.

In summary, to optimize the diagnostic yield of HVEM for patients with DRE, a substantially longer monitoring duration than presently in use is needed. Future technical advancements and prospective studies in specific populations of patients are required to allow a larger-scale use of this important diagnostic tool.

## Data availability statement

The original contributions presented in the study are included in the article/Supplementary material, further inquiries can be directed to the corresponding author/s.

## Author contributions

TV conceptualized the study, drafted the manuscript, and critically read the manuscript. TS contributed to the simulation code writing and critically read the manuscript. DD drafted the manuscript and critically read the manuscript. DEl critically read the manuscript. TG critically read the manuscript. MM conceptualized the study, wrote the simulation code, drafted the manuscript, and supervised its final version. DEk conceptualized the study, contributed to the simulation code writing and wrote the final version of the manuscript. All authors contributed to the article and approved the submitted version.

## Conflict of interest

Authors MM and DEk are the inventors of patents related to HVEM development. Authors DEl, MM, and DEk are involved with VIRDA startup company, which develops HVEM systems. The remaining authors declare that the research was conducted in the absence of any commercial or financial relationships that could be construed as a potential conflict of interest.

## Publisher's note

All claims expressed in this article are solely those of the authors and do not necessarily represent those of their affiliated organizations, or those of the publisher, the editors and the reviewers. Any product that may be evaluated in this article, or claim that may be made by its manufacturer, is not guaranteed or endorsed by the publisher.

## References

[1] Binnie CD Rowan AJ Overweg J Meinardi H Wisman T Kamp A. Telemetric EEG and video monitoring in epilepsy. Neurology. (1981) 31:298–303. 10.1212/WNL.31.3.298 7193821

[2] Yen DJ Chen C Shih YH Guo YC Liu LT Yu HY. Antiepileptic drug withdrawal in patients with temporal lobe epilepsy undergoing presurgical video-EEG monitoring. Epilepsia. (2001) 42:251–5. 10.1046/j.1528-1157.2001.15100.x 11240598

[3] Slater JD Eaddy M Butts CM Meltser I Murty S. The real-world economic impact of home-based video electroencephalography: the payer perspective. J Med Econ. (2019) 22:1030–40. 10.1080/13696998.2019.1636382 31237168

[4] Fahoum F Omer N Kipervasser S Bar-Adon T Neufeld M. Safety in the epilepsy monitoring unit: a retrospective study of 524 consecutive admissions. Epilepsy Behav. (2016) 61:162–7. 10.1016/j.yebeh.2016.06.002 27351727

[5] Sylaja PN Radhakrishnan K. Problems and pitfalls in developing countries. Epilepsia. (2003) 44 Suppl 1:48–50. 10.1046/j.1528-1157.44.s.1.11.x 12558833

[6] Dash D Hernandez-Ronquillo L Moien-Afshari F Tellez-Zenteno JF. Ambulatory EEG: a cost-effective alternative to inpatient video-EEG in adult patients. Epileptic Disord. (2012) 14:290–7. 10.1684/epd.2012.0529 22963900

[7] Biondi A Santoro V Viana PF Laiou P Pal DK Bruno E. Noninvasive mobile EEG as a tool for seizure monitoring and management: a systematic review. Epilepsia. (2022). 10.1111/epi.17220 35271736 PMC9311406

[8] Klein H Pang T Slater J Ramsay RE. How much time is enough? Establishing an optimal duration of recording for ambulatory video. EEG Epilepsia Open. (2021) 6:569–78. 10.1002/epi4.12517 34197695 PMC8408602

[9] Struck AF Cole AJ Cash SS Westover MB. The number of seizures needed in the EMU. Epilepsia. (2015) 56:1753–9. 10.1111/epi.13090 26222350 PMC4877132

[10] Tatum WO Mani J Jin K Halford JJ Gloss D Fahoum F. Minimum standards for inpatient long-term video-electroencephalographic monitoring: a clinical practice guideline of the International League Against Epilepsy and International Federation of Clinical Neurophysiology. Epilepsia. (2022) 63:290–315. 10.1111/epi.16977 34897662

[11] Ferastraoaru V Goldenholz DM Chiang S Moss R Theodore WH Haut SR. Characteristics of large patient-reported outcomes: where can one million seizures get us? Epilepsia Open. (2018) 3:364–73. 10.1002/epi4.12237 30187007 PMC6119749

[12] Leguia MG Andrzejak RG Rummel C Fan JM Mirro EA Tcheng TK. Seizure cycles in focal epilepsy. JAMA Neurol. (2021) 78:454–63. 10.1001/jamaneurol.2020.5370 33555292 PMC7871210

[13] Karoly PJ Goldenholz DM Freestone DR Moss RE Grayden DB Theodore WH. Circadian and circaseptan rhythms in human epilepsy: a retrospective cohort study. Lancet Neurol. (2018) 17:977–85. 10.1016/S1474-4422(18)30274-6 30219655

[14] Elger CE Hoppe C. Diagnostic challenges in epilepsy: seizure under-reporting and seizure detection. Lancet Neurol. (2018) 17:279–88. 10.1016/S1474-4422(18)30038-3 29452687

[15] King-Stephens D Mirro E Weber PB Laxer KD Van Ness PC Salanova V. Lateralization of mesial temporal lobe epilepsy with chronic ambulatory electrocorticography. Epilepsia. (2015) 56:959–67. 10.1111/epi.13010 25988840 PMC4676303

[16] Syed TU LaFrance WC Loddenkemper T Benbadis S Slater JD El-Atrache R. Outcome of ambulatory video-EEG monitoring in a 10,000 patient nationwide cohort. Seizure. (2019) 66:104–11. 10.1016/j.seizure.2019.01.018 30910235

[17] Chen-Block S Abou-Khalil BW Arain A Haas KF Lagrange AH Gallagher MJ. Video-EEG results and clinical characteristics in patients with psychogenic nonepileptic spells: the effect of a coexistent epilepsy. Epilepsy Behav. (2016) 62:62–5. 10.1016/j.yebeh.2016.06.018 27450307

[18] Shad EHT Molinas M Ytterdal T. Impedance and noise of passive and active dry EEG electrodes: a review. Ieee Sens J. (2020) 20:14565–77. 10.1109/JSEN.2020.3012394

[19] Duun-Henriksen J Baud M Richardson MP Cook M Kouvas G Heasman JM. A new era in electroencephalographic monitoring? Subscalp devices for ultra-long-term recordings. Epilepsia. (2020) 61:1805–17. 10.1111/epi.16630 32852091

[20] Stirling RE Maturana MI Karoly PJ Nurse ES McCutcheon K Grayden DB. Seizure forecasting using a novel sub-scalp ultra-long term EEG monitoring system. Front Neurol. (2021) 12:713794. 10.3389/fneur.2021.713794 34497578 PMC8419461

[21] Herzog AG Klein P Ransil BJ. Three patterns of catamenial epilepsy. Epilepsia. (1997) 38:1082–8. 10.1111/j.1528-1157.1997.tb01197.x 9579954

