# Supplementary material for: What is the optimal duration of home-video-EEG monitoring for patients with <1 seizure per day? A simulation study
Source: Front Neurol. 2022 Aug 22;13:938294. doi: 10.3389/fneur.2022.938294 (PMC9441894; doi:10.3389/fneur.2022.938294)
Supplement: Supplementary file 1 [file Data_Sheet_1.docx]

Table S1

The point probability densities corresponding to the chosen points on X-axis of Figure 1S from Ferastoauru et al, 2018:

| Seizures/month (X-axis) | Point probability density: adults (Y-axis) | Point probability density: children (Y-axis) |
| --- | --- | --- |
| 1 | 0.160 | 0.090 |
| 2 | 0.130 | 0.100 |
| 3 | 0.100 | 0.080 |
| 4 | 0.075 | 0.070 |
| 5 | 0.060 | 0.060 |
| 6 | 0.050 | 0.045 |
| 7 | 0.040 | 0.040 |
| 8 | 0.035 | 0.035 |
| 9 | 0.032 | 0.032 |
| 10 | 0.025 | 0.028 |
| 15 | 0.020 | 0.023 |
| 20 | 0.012 | 0.015 |
| 25 | 0.008 | 0.010 |
| 30 | 0.006 | 0.008 |

**Table S2. Percentages of adult patients reaching HVEM goals**

| Weeks | Patients until recording of the first seizure | Patients until recording of 3 seizures in different days | Patients until recording of 5 seizures in different days |
| --- | --- | --- | --- |
| 1 | 68.04% | 23.43% | 5.11% |
| 2 | 83.18% | 47.40% | 26.28% |
| 3 | 90.23% | 61.32% | 41.08% |
| 4 | 93.81% | 70.45% | 51.35% |
| 5 | 95.92% | 77.28% | 59.18% |
| 6 | 97.37% | 82.23% | 65.43% |
| 7 | 98.25% | 86.05% | 70.61% |
| 8 | 98.78% | 89% | 74.91% |
| 9 | 99.14% | 91.31% | 78.59% |
| 10 | 99.43% | 93.24% | 81.57% |
| Mean (days) | 7.50 | 24.22 | 40.66 |
| Median (days) | 3 | 15 | 26 |
| Standard deviation (days) | 12.07 | 25.67 | 38.03 |

**Table S3. Percentages of pediatric patients reaching HVEM goals**

| Weeks | Patients until recording of the first seizure | Patients until recording of 3 seizures in different days | Patients until recording of 5 seizures in different days |
| --- | --- | --- | --- |
| 1 | 73.82% | 29.09% | 6.43% |
| 2 | 87.20% | 55.44% | 32.80% |
| 3 | 92.75% | 68.59% | 48.86% |
| 4 | 95.59% | 76.95% | 59.36% |
| 5 | 97.19% | 82.75% | 67.03% |
| 6 | 98.19% | 86.93% | 72.92% |
| 7 | 98.81% | 89.94% | 77.42% |
| 8 | 99.22% | 92.13% | 81.11% |
| 9 | 99.47% | 93.78% | 84.05% |
| 10 | 99.63% | 95.10% | 86.50% |
| Mean (days) | 6.07 | 20.26 | 34.40 |
| Median (days) | 2 | 11 | 21 |
| Standard deviation (days) | 10.42 | 22.97 | 34.29 |
